# RNAs as Candidate Diagnostic and Prognostic Markers of Prostate Cancer—From Cell Line Models to Liquid Biopsies

**DOI:** 10.3390/diagnostics8030060

**Published:** 2018-08-30

**Authors:** Marvin C. J. Lim, Anne-Marie Baird, John Aird, John Greene, Dhruv Kapoor, Steven G. Gray, Ray McDermott, Stephen P. Finn

**Affiliations:** 1 Department of Histopathology and Morbid Anatomy, Trinity Translational Medicine Institute, Trinity College Dublin, Dublin D08 W9RT, Ireland; LIMMC@tcd.ie (M.C.J.L.); AIRDJ@tcd.ie (J.A.); GREENEJO@tcd.ie (J.G.); DHRUVKAPOOR@rcsi.ie (D.K.); 2 Department of Medical Oncology, Tallaght University Hospital, Dublin D24 NR0A, Ireland; RAY.MCDERMOTT@amnch.ie; 3 Cancer and Ageing Research Programme, Queensland University of Technology, Brisbane, QLD 4000, Australia; BAIRDA@tcd.ie; 4 Department of Clinical Medicine, Trinity College Dublin, College Green, Dublin D02 PN40, Ireland; SGRAY@stjames.ie; 5 Thoracic Oncology Research Group, Labmed Directorate, St. James’s Hospital, Dublin 08 W9RT, Ireland; 6 School of Biological Sciences, Dublin Institute of Technology, Dublin D08 NF82, Ireland; 7 Department of Medical Oncology, St. Vincent’s University Hospital, Dublin D04 YN26, Ireland; 8 Department of Histopathology, St. James’s Hospital, P.O. Box 580, James’s Street, Dublin D08 X4RX, Ireland

**Keywords:** RNA, mRNA, ncRNA, lncRNA, circRNA, miRNA, snoRNA, sdRNA, tRNA, tRF, biomarker, prostate cancer

## Abstract

The treatment landscape of prostate cancer has evolved rapidly over the past five years. The explosion in treatment advances has been witnessed in parallel with significant progress in the field of molecular biomarkers. The advent of next-generation sequencing has enabled the molecular profiling of the genomic and transcriptomic architecture of prostate and other cancers. Coupled with this, is a renewed interest in the role of non-coding RNA (ncRNA) in prostate cancer biology. ncRNA consists of several different classes including small non-coding RNA (sncRNA), long non-coding RNA (lncRNA), and circular RNA (circRNA). These families are under active investigation, given their essential roles in cancer initiation, development and progression. This review focuses on the evidence for the role of RNAs in prostate cancer, and their use as diagnostic and prognostic markers, and targets for treatment in this disease.

## 1. Introduction

Prostate cancer is the second most common invasive cancer in men globally [1]. In Western Europe and the United States (US), it is the second leading cause of male cancer-associated mortality [2]. Approximately 79% of patients with prostate cancer are diagnosed at a localized stage based on the Surveillance, Epidemiology and End Results Program (SEER) data [3]. Therapeutic options such as radical prostatectomy, external beam radiotherapy (EBRT) or brachytherapy are primarily recommended for men with localized curative prostate cancer [4]. Despite initial curative effort by radical prostatectomy for localized disease, approximately 35% of men will experience a biochemical recurrence [5], and a higher recurrence rate of 40–60% has been witnessed after EBRT or brachytherapy [6,7]. Androgen deprivation therapy (ADT) is the standard of treatment for metastatic disease [2], with an average initial response of approximately 18 months, however resistance inevitably develops resulting in castration-resistant prostate cancer (CRPC), which is currently incurable [8].

The treatment paradigm of prostate cancer has evolved rapidly in the last decade due to greater availability and choice of therapeutic agents. Docetaxel chemotherapy was approved in 2004 for treatment of patients with CRPC after being the first agent that successfully showed improvement in overall survival [9]. However, there was a paucity of survival prolonging therapies for this cohort of patients until 2010. Since then, five new treatments that improve overall survival of patients with CRPC have been added to the inventory, consisting of novel second generation antiandrogens (Abiraterone and Enzalutamide), chemotherapy (Cabazitaxel), radionuclide therapy (Alpha-radium 223) and immunotherapy (Sipuleucel-T) (Table 1). Despite the effectiveness of these new agents, a proportion of patients will have no response, termed intrinsic resistance, and all patients will eventually acquire secondary resistance resulting in disease progression. For instance, approximately 20% to 40% of patients will have intrinsic resistance to a novel second generation antiandrogen [10,11,12].

Additionally, the treatment landscape of de novo metastatic castration-sensitive prostate cancer (CSPC) has recently been refashioned by the findings of the CHAARTED [13] and LATITUDE [14] studies, which showed that concomitant upfront Docetaxel or Abiraterone respectively, at the beginning of hormonal therapy improved overall survival compared to hormonal therapy alone. These studies highlighted the importance of appropriate sequencing of treatment, in optimizing clinical benefit and patient outcome. Thus, even though we currently have an impressive therapy arsenal, there is a lack of effective clinical tools centred on patient selection and treatment sequencing. Without such tools, there is a difficulty providing the optimal therapy for each patient at each point in their care pathway.

**Figure 1:**
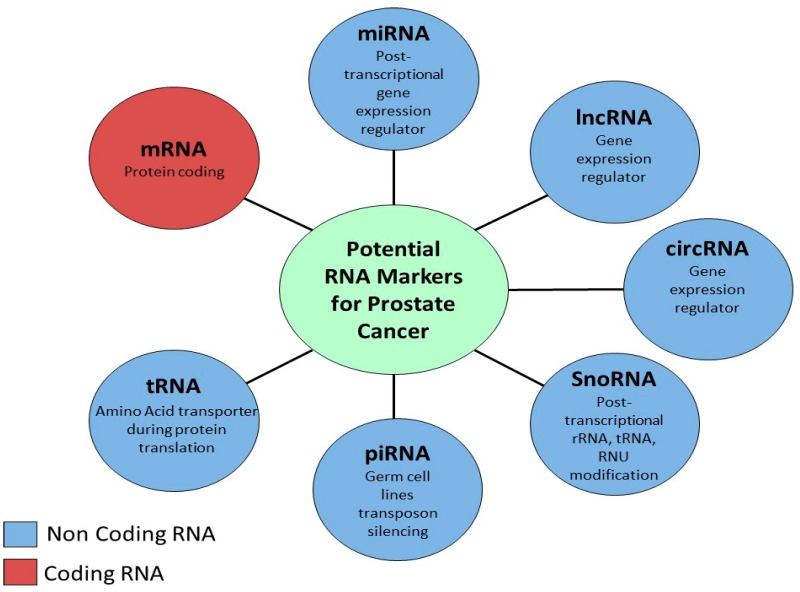
Types of RNAs and their associated function.

Nevertheless, the advent of next-generation sequencing (NGS) has furthered our understanding of the genomic and transcriptomic architecture of prostate and other cancers [15,16,17], which has resulted in novel molecular biomarkers strategies. Recently there has been a resurgence of interest in the role of non-coding RNA (ncRNA) in prostate cancer biology. ncRNA consists of several different classes including sncRNA and lncRNA (Figure 1), both of which have been studied widely [18]. Another recently discovered type of ncRNA, called circular RNA (circRNA), appears to have important roles in cancer initiation, development and progression [19,20]. This review focuses on the evidence in the literature for the role of RNAs in prostate cancer, and their use as diagnostic and prognostic markers, and targets for treatment in this disease.

## 2. Current Diagnostic, Prognostic and Predictive Strategies

Prostate-specific antigen (PSA) has transformed prostate cancer diagnostics since its introduction as a serum tumor marker in 1987 [25]. It is still the only universally utilized biomarker to determine whether prostate biopsies would be appropriate and also aid in the monitoring of treatment response in addition to its most contentious role, screening for the disease itself [26,27,28]. Regarding its use in screening, it may lead to overtreatment of clinically indolent disease resulting in poor quality of life [29,30]. This is due to the scarcity of a useful screening tool to distinguish between indolent and aggressive disease [31]. PSA is not an ideal biomarker due to poor tumor specificity and its level can be influenced by many factors, such as trauma, prostatitis, age, and concomitant medication [32,33,34]. Furthermore, there is no definite cut-off PSA value which guarantees a negligible risk of harboring prostate cancer. Thompson et al. demonstrated that 15.2% of men with “normal” PSA level (cut-off of ≤4 ng/mL) were at risk for prostate cancer and 14.9% of these men had a high Gleason grade disease [35]. Only 25–30% of patients with PSA levels above 4 ng/mL were positive for the presence of cancer on tissue biopsy [36]. Patients are subjected to a high number of unwarranted successive biopsies owing to high false positive rate due to a low PSA specificity of only 12.8% when the cut-off value of 4 ng/mL is used [37]. An ideal PSA cut-off point with both high sensitivity and specificity for prostate cancer diagnosis does not exist; however, many endeavors to increase its diagnostic power have been undertaken. The Prostate Health Index (PHI), which integrates PSA sub-forms into a diagnostic score showed superior performance in detecting prostate cancer compared to PSA testing alone [38]. Risk stratification is crucial in providing optimal treatment of prostate cancer. Risk group stratification which incorporated stage, grade and PSA has been extensively validated and provides better information for treatment recommendations compared to staging alone [39]. The Tumor Node Metastasis (TNM) system is used to determine the clinical stage and correlates with overall survival [40]. The Gleason grade delineates the morphological characteristics of prostate carcinoma, which correspond with clinical behavior [41]. The International Society of Urological Pathology (ISUP) introduced a new prostate cancer grading system in 2014 that assigns Gleason grade group from 1 to 5 (very low, low, intermediate, high and very high), which predicts the risk of recurrence after primary treatment, and has been validated in a few studies [42,43,44]. This Gleason grade group system is superior to Gleason score in terms of informing patients of their actual risk level and may prevent unnecessary treatment [42]. Apart from using individual prognostic variables, a nomogram, which is a tool that combines the relevant prognostic parameters to generate more accurate results, can be used. A widely used nomogram, the Partin tables, were the first to be widely used by urologists to counsel men with clinically localized prostate cancer for the last few decades [45,46,47]. Other nomograms have also been developed according to the needs of a specific clinical setting such as assessing the risk of biochemical progression post curative therapy or suitability for active surveillance, however none has perfect prognostic precision [48,49].

Additionally, the improvement in genetic analysis has led to increased recognition of genetic mutations in DNA repair pathways such as mismatch repair (MMR) and homologous recombination (HR) in prostate cancer [50]. Approximately 15–25% of patients with CRPC harbor somatic DNA repair gene mutations involving *BRCA 1* or *BRCA 2* and *ATM* [15]. These cohorts of patients have been shown to benefit from Olaparib, which is a type of Poly (ADP-ribose) polymerase (PARP) inhibitor [51] and platinum-containing chemotherapy [52]. Thus, these genetic mutations play an essential role as predictive biomarkers when determining treatment options. The flaws in the current diagnostic, prognostic and predictive strategies reflect the importance of molecular biomarkers in tailoring the treatment of patients with prostate cancer.

## 3. Candidate RNAs as Biomarkers for Prostate Cancer

### 3.1. Messenger RNAs (mRNAs) as Biomarkers for Prostate Cancer

mRNA is a type of RNA, which is translated into protein through the ribosomal machinery. mRNA can serve a role as a biomarker, and has been well studied in many different cancers [53,54]. In 2012, March-Villalba et al. reported that circulating plasma Telomerase Reverse Transcriptase (*hTERT*) mRNA is a potentially helpful diagnostic biomarker for prostate cancer, with a sensitivity of 85% and specificity of 90%; which compares favorably to serum PSA, which has sensitivity of 83% and specificity of 47% [55]. The study examined the plasma *hTERT* mRNA level of 105 patients with an elevated PSA level of >4 ng/mL and 68 normal controls. The patients were stratified according to histopathological criteria into prostate cancer, prostatitis, benign prostatic hyperplasia (BPH) or normal cohort. Circulating *hTERT* mRNA was an independent predictor of prostate cancer and also a significant prognostic biomarker for biochemical recurrence [55]. Haldrup et al. analyzed *SLC18A2* gene expression in prostate cancer in terms of promoter methylation, mRNA and protein expression [56]. A cohort of 412 prostate cancer tissues and 45 benign prostate tissues were used to assess *SLC18A2* mRNA expression. The group found that *SLC18A2* mRNA expression levels were significantly decreased in prostate cancer samples, with low expression levels significantly associated with PSA recurrence after radical prostatectomy (multivariate hazard ratio (HR) 0.13, *p* < 0.05) suggestive of independent prognostic value [56]. Danila et al. further investigated a panel of circulating mRNA (*KLK3*, *KLK2*, *HOXB13*, *GRHL2* and *FOXA1*) using RT-PCR in 97 patients with metastatic CRPC [57]. The study reported that the gene panel could predict overall survival based on the number of transcripts detected, i.e., ≥2 (detectable) or <2 (undetectable) with median survival for ‘detectable’ was 11.2 months and ‘undetectable’ was 29.2 months [57]. In another study, an expression panel of 31 mRNAs from tumor samples, was developed by Cuzick et al. relating to cell cycle progression such as *FOXM1*, *CDC20*, *CDKN3*, *KIF11*, and *PLK1*, which are then converted to cell cycle progression score, CCP [58]. The data demonstrated that the CCP score was significantly associated with PSA recurrence over a decade follow up period. Bishoff et al. further showed that a high CCP score from tumor biopsy samples of men with prostate cancer who had radical prostatectomy corresponded with increased biochemical recurrence rate, metastatic development or mortality from prostate cancer [59]. Klein et al. analyzed the expression of 732 genes, in tissue specimens from 441 patients who underwent a radical prostatectomy, using qRT-PCR and identified a panel of 228 genes, which were related with biochemical recurrence irrespective of Gleason pattern [60]. From the panel of 228 genes, 73 genes were selected in addition to a further 8 genes. These genes are associated with recurrence, prostate cancer death and adverse pathology, and were evaluated in the biopsy cohort (*n* = 167). A final total of 12 cancer-related genes linked to four different molecular pathway; stromal response (*BGN*, *SFRP4*, *COL1A1*), androgen (*KLK2*, *FAM13C*, *SRD5A2*, *AZGP1)*, cellular organization (*TPM2*, *FLNC*, *GSN*, *GSTM2*), and proliferation (*TPX2*) together with five reference genes (*ARF1*, *ATP5E*, *CLTC*, *GPS1*, *PGK1*) were selected to calculate the genomic prostate cancer score (GPS) known as the Oncotype DX test [60]. The GPS values range from 0 to 100, with a higher score reflecting more aggressive disease. This score was validated in a prospective cohort of 395 patients who had low to intermediate clinical risk suitable for active surveillance. The GPS was able to predict high grade tumor (odds ratio (OR) per 20 GPS units: 2.3; 95% CI: 1.5–3.7; *p* < 0.001) and high stage disease (OR per 20 GPS units: 1.9; 95% CI: 1.3–3.0; *p* = 0.003). The group reported that GPS improves risk stratification for patients with localized prostate cancer which will help to guide appropriate management of disease [60]. Cullen et al. evaluated the risk of biochemical recurrence in a cohort of 402 patients with low or intermediate prostate cancer [61]. The author found that GPS is capable of predicting biochemical recurrence (HR univariate per 20 GPS units: 2.9; *p* < 0.001) as well as time to metastases (HR per 20 units; 3.8; *p* = 0.032) [61]. Multigene mRNA expression profile have been successfully applied clinically in other malignancies such as the Oncotype DX test in breast cancer [62], however the European Association of Urology (EAU) have yet to make a concrete recommendation on Oncotype DX in prostate cancer. Guidelines on use are pending on results from prospective multi-center trials (ClinicalTrials.gov Identifier: NCT03502213) prior to large scale practice-changing utilization [63].

### 3.2. MicroRNAs (miRNAs) as Biomarkers for Prostate Cancer

A large proportion of the human genome (70–90%) is transcribed into RNA, however most RNA transcripts are non-coding and only 2% of the genome encodes for protein [64]. Non-coding RNAs are starting to gain recognition as one of the key mediators in gene regulation [65,66,67]. These vital players can generally be categorised into either sncRNAs (<200 nucleotides long) which includes miRNA, small nucleolar RNA (snoRNA), piwi-interacting RNA (piRNA), or lncRNAs (>200 nucleotides long), and circRNA [65,66,67]. miRNA is classified as sncRNA, consisting of approximately 22 nucleotides in length with the ability to regulate genes post-transcriptionally [68]. miRNA originates from primary transcript (pri-miRNA), which is initially processed into approximately 70 nucleotides long hair pin precursor miRNA (pre-miRNA) by cellular RNase III (Drosha) and double stranded RNA-binding domain protein (DGCR8) [69]. Another RNase III (Dicer), then cleaves the pre-mRNA to form duplex strands termed miRNA-5p and miRNA-3p, whereby the ‘5p’ and ‘3p’ are named according to the 5′- or 3′-arm of the original transcript [69]. miRNAs can exhibit affinity to bind to 3′ untranslated region (UTR) of mRNA or to 5′ UTR of mRNA leading to direct mRNA cleavage and translational repression [70]. Besides gene repression, miRNA can directly or indirectly activate gene expression [71]. Through these regulatory functions, miRNA is involved in cellular differentiation, proliferation, migration and apoptosis [72]. miRNA can be considered as either an Onco-miR, if it inhibits tumor suppressor genes; or a tumor suppressor-miR if it inhibits proto-oncogenes [73]. These critical functions have led to extensive investigations into the capacity of miRNAs to act as clinically relevant biomarkers in prostate cancer for the past decade [74] (Table 2).

In 2006, Volina et al. [75] first observed a general trend of up-regulated miRNA expression in prostate cancer, which was later supported by Ambs et al. in 2008 [76]. Song et al. further validated this general up-regulated trend by using NGS in 2015 [77]. In contrast, several studies observed a general down-regulation of miRNA expression in prostate cancer compared to the normal prostate [78,79]. This discrepancy in expression between studies may be linked to small sample size or variation in technical protocols. In 2010, Schaefer et al. reported miR-96 expression to be predictive of biochemical recurrence in patients post radical prostatectomy after examining a 79-patient tumor sample set. The group also demonstrated that tumors with a high level of miR-96 had decreased recurrence-free survival [80]. Additionally, Haflidadottir et al. showed that high miR-96 expression level corresponded with poor overall survival after radical prostatectomy and also higher grade tumor [81]. The group also investigated the miRNA-mRNA pathway using prostate cancer cell lines and demonstrated that miR-96 could bind *FOXO1* mRNA, which is a tumor suppressor, resulting in a reduction of FOXO1 protein thus, increasing cell growth and proliferation [81].

In 2012, Martens-Uzunova et al. utilized NGS and miRNA microarray, with results of both platforms cross validated by qRT-PCR, to analyze miRNA expression in 102 fresh frozen samples from patients with prostate cancer disease progression [82]. The group constructed a miR predictor consisting of 25 miRNAs, which could identify good prognosis and poor prognosis subgroups, comprising 13 miRNA that were down-regulated and 12 that were up-regulated [82]. The same group examined an additional set of 49 prostate cancer tissues and 25 normal controls for the presence of 13 preselected miRNAs. Four miRNAs (miR-96-5p, miR-183-5p, miR-145-5p, miR-221-5p) were selected to construct a miR index quote (miQ) based on the best diagnostic discrimination whereby two were up-regulated (miR-96-5p and miR-183-5p) and two were down-regulated (miR-145-5p and miR-221-5p). The group reported that the miQ score had good capability for diagnosing prostate cancer with high accuracy (Area under the curve (AUC): 0.931) and predict tumor aggressiveness (AUC: 0.895), metastatic status (AUC: 0.827) and overall survival (*p* = 0.0013, Wilcoxon test HR = 6.5, median survival 2 vs. 5 years) [83]. These biomarker abilities of the miQ score can be explained by the function of the miRNAs it is comprised of. miR-96 can mediate cell proliferation and cell growth through FOXO1 inhibition in prostate cancer [81] and miR-183 can regulate *PTEN*, which is a known tumor suppressor in prostate cancer [84]. Overexpression of miR-145 in PC3 prostate cancer cells has been reported to cause cycle arrest and apoptosis as a result of upregulating the pro-apoptotic gene, *TNFSF10* [85]. miR-145 was also found to inhibit epithelial-mesenchymal transition (EMT), suggesting that a reduction of miR-145 may trigger a metastatic cascade [86]. Spahn et al. reported that downregulation of miR-221 is correlated with upregulation of *c-Kit* [87] and two other studies have shown the potential of *c-Kit* involvement in prostate cancer bone metastasis progression [88,89]. Zheng et al. in 2014 further showed that low miR-221 (part of the miQ score) levels are associated with a higher biochemical recurrence rate after radical prostatectomy [90], in addition to its association with prostate cancer diagnosis [82,83]. However, some other studies have demonstrated conflicting results. Yaman et al. reported significantly higher expression of miR-221 in blood plasma of patients with metastatic prostate cancer compared to localized disease patients [91]. miR-221 is associated with metastasis formation, thus in conjunction with miR-141 could potentially be used to detect men with bone metastasis from those with localized disease (AUC: 0.755) [91]. Furthermore, Sun et al. found significant expression of miR-221 in CRPC cell lines and tissue [92,93]. The study showed that up-regulation of miR-221 decreases HECTD2 or RAB1A which promotes androgen independent prostate cancer cell growth [94].

Mitchell et al. in 2008 reported that miR-141, which is up-regulated in prostate cancer tissue, is also up-regulated and stable in the serum of patients with prostate cancer [95]. This significantly added to the value of miRNA being a potential non-invasive biomarker. Many studies evaluating the potential of circulating miRNA as a biomarker have been performed. Few plasma or serum miRNAs expression have been consistently shown to be increased in patients with prostate cancer such as miR-141, miR-375 and miR-21. Brase et al. screened 667 miRNAs in serum samples of 7 patients with metastatic disease and 14 patients with localized prostate cancer. A panel of 5 miRNAs (miRNA-375, miRNA-9, miRNA-141, miRNA-200b and miRNA-516a-3p) were then further validated in 71 patients with prostate cancer [96]. Circulating miR-141 and miR-375 expression were highly correlated with a high risk tumor such as high Gleason score or lymph node positive disease, which reflects their potential as good prognostic markers [96]. The group further examined the expression of these miRNAs in 36 prostate cancer tissues compared to 36 benign prostate tissues. The expression of both miR-141 and miR-375 was highly up-regulated in prostate cancer tissues compared to normal controls [96]. miR-141 is part of the miR-200 family, which is associated with the regulation of EMT [97]. Recently, Selth et al. reported a novel pathway of miRNA regulation in prostate cancer where a ZEB1-miR-375-YAP1 regulatory circuit, controls epithelial plasticity and cancer cell invasion [98]. The group found that miR-375 can act as a tumor suppressor inhibiting EMT in contrast to previous studies where miR-375 was associated with poor prognosis and high risk tumor [98]. This would suggest miR-375 has a dual role in prostate cancer progression, which is a new concept reported by Costa-Pinheiro et al. [99]. The group demonstrated that the 22Rv1 prostate cancer cell line expressed high level of miR-375, whereas PC-3 prostate cancer cell lines have significantly lower expression. Interestingly, 22Rv1 cell viability was reduced when miR-375 was downregulated using anti-miR-375, however PC-3 cell viability was reduced when miR-375 was overexpressed [99]. Shen J et al. further examined the plasma of 82 patients with prostate cancer, whereby the group identified that the expression levels of miR-20a, miR-21, miR-145 and miR-221 could detect high risk patients from low risk patients (AUC: 0.824) [100]. miR-20a exhibits an anti-apoptotic effect through modulation of *E2F2* and *E2F3*, which are part of the E2F family of transcription factors, regulating cell cycle and apoptosis [101]. miR-21 upregulation was shown to promote angiogenesis by targeting PTEN resulting in activation of AKT and ERK1/2 pathways leading to overexpression of HIF-1α and *VEGF* [102]. Downregulation of miR-145 has been reported in many prostate cancer studies using tumor samples [78,79,80,83] which is in contrast to the study reported by Shen J et al., however this group utilized plasma instead [100]. The reason for this discrepancy could be due to different sample types.

Apart from blood-based miRNA levels, Bryant et al. reported that urine of men with prostate cancer have significantly higher miR-107 and miR-574-3p compared to a healthy cohort, indicating that circulating miRNA can be tested in various bodily fluids [103]. Since combining a panel of circulating miRNA has been suggested to provide better sensitivity and specificity [100,104], De Souza et al. investigated the combination of circulating mRNA and miRNA as a biomarker panel for prostate cancer [105]. A final selection of 11 genes and eight miRNAs expressed between normal and tumor samples were selected for validation from a total of 2267 genes and 49 miRNAs respectively. These genes and miRNAs were then validated in 102 patients with prostate cancer and 50 normal controls. The group reported that two genes, *OR51E2* (AUC: 0.65) and *SIM2* (AUC: 0.61); and two miRNAs, miR-200b (AUC: 0.57) and miR-200c (AUC: 0.62) showed a significant association with prostate cancer. Both *OR51E2* and *SIM2* had a remarkable sensitivity of 100%, but lower specificity for *OR51E2* (50%) and *SIM2* (72%) in distinguishing prostate cancer from normal despite PSA < 4 ng/mL [105]. High miR-200b expression is associated with high PSA of >10 ng/mL, bilateral tumor and bone metastasis, whereas high miR200c expression correlates with high Gleason score [105]. The combination of all four transcripts showed a sensitivity of 67% and a specificity of 75% in diagnosing prostate cancer [105].

### 3.3. Long Non-Coding RNAs (lncRNAs) 

lncRNA is a group of non-coding RNA > 200 nucleotides in length. So far 105,255 lncRNAs have been identified in the human genome [106]. lncRNAs play a role in gene expression regulation affecting transcription and translation through chromatin complex modulations [107,108]. In cancer, lncRNA may promote cell proliferation, invasion and progression, inducing angiogenesis and facilitating resistance to apoptosis [109]. Many lncRNA exhibit cell type-specific expression patterns and have both nuclear and cytoplasmic localization [107,110]. Given that lncRNA are present in bodily fluids such as urine, blood and serum and are specifically expressed at different stages of prostate cancer [111,112], they may be a good candidate biomarker.

PCA3 (Prostate Cancer Antigen 3) was discovered in 1999 and has very much been the primary focus of study as a prostate biomarker [113,114]. The PCA3 level was increased approximately 100-fold in prostate cancer tissue compared to paired normal prostate tissues [114]. Furthermore, PCA3 knockdown led to an up-regulation of epithelial markers E-cadherin, claudin-3 and CK18 and down-regulation of mesenchymal marker vimentin, which suggests its involvement in EMT [115]. Besides, it may also inhibit androgen receptor (AR) signaling, cell growth and viability [115]. Additionally, PCA3 regulates the expression of genes associated with angiogenesis, signal transduction and apoptosis [115]. PCA3 can also regulate miR-1261 by acting as a miRNA sponge when the transcription factor Snail binds to its promoter region. This in turn, reduces miR-1261, resulting in increased expression of *PRKD3* [116]. The high *PRKD3* expression can promote invasion and metastatic progression of prostatic cancer [116]. Another study showed that knockdown of PCA3 sensitized prostate cancer cells to Enzalutamide [117], thus PCA3 may act as both a potential diagnostic and therapeutic biomarker. Urinary PCA3 diagnostic performance has been validated in a multicentre study of 534 men showing a sensitivity of 65% and specificity of 66% compared to serum PSA, which has the same sensitivity (65%) but lower specificity (47%) [118]. Urinary PCA3 assay (Progensa) was approved by the Food and Drug Administration (FDA) in 2012 for men above 50 years of age of whom a subsequent biopsy is otherwise warranted after previous negative biopsies [119]. Urine from post digital rectal examination is used for the Progensa assay and a PCA3 score is obtained from the ratio of PCA3 RNA to PSA RNA detected. PCA3 score of <25 is considered low risk of harboring prostate cancer whereas a PCA3 score of ≥25 is considered high risk with more than 25% chance of a positive biopsy result [120]. The sensitivity and specificity depend on the cut off value of the PCA3 score. For instance, Leyton et al. showed that a PCA3 score of 25 would provide a sensitivity of 82% and specificity of 50.8%, however with a PCA3 score of 35 the sensitivity would decrease to 68.4% but specificity would increase to 58.3% [111]. Recently, Cui Y et al. performed a systemic review and meta-analysis of 46 clinical trials consisting of a total of 12,295 patients which used urinary PCA3 for diagnosing prostate cancer [121]. The group reported that the pooled sensitivity was 0.65 (95% CI: 0.63–0.66) and specificity was 0.73 (95% CI: 0.72–0.74) [121]. Although PCA3 has improved specificity, it is not sensitive enough as a standalone diagnostic biopsy decision tool. Thus, Tomlin SA et al. evaluated the combination of urinary PCA3 with urinary Transmembrane protease, serine 2 (*TMPRSS2*): ETS related gene (*ERG*)to aid in diagnosis and biopsy decision making. The group evaluated 1244 men and showed that combination of *TMPRSS2*:*ERG* plus PCA3 is superior in diagnosing prostate cancer having AUC of 0.751 compared to PSA plus PCA3 (AUC: 0.726) and PSA alone (AUC: 0.585) [122].

Metastasis associated lung adenocarcinoma transcript 1 (MALAT 1), a lncRNA initially investigated in lung cancer, is also expressed in prostate cancer tissues and cell lines, with the expression level associated with high Gleason score, PSA score, and tumor size reflective of a poor risk subgroup [123]. Furthermore, the level of MALAT 1 increases during the transition from castration-sensitive to castration-resistance as reported by Ren et al. [123]. Thegroup also found that MALAT 1 suppression in cell lines led to a reduction in cell growth and invasion as well as inducing cell cycle arrest and promoting apoptosis [123]. When suppressed in prostate cancer xenografts, in castrated nude mice, it reduced tumour growth and metastatic progression thus demonstrating its capability of acting as a potential therapeutic target [123]. The same group examined MALAT 1 derived from plasma (MD-miniRNA) as a means to detect prostate cancer. They demonstrated levels were significantly raised in 196 patients with prostate cancer and had a sensitivity of 58.6% and specificity of 84.8% in distinguishing a patient with prostate cancer from a healthy control making it a potential non-invasive diagnostic biomarker [112]. The diagnostic accuracy to distinguish patients with prostate cancer compared to healthy cohort was further demonstrated by Xue et al. showing AUC 0.86 (95% CI: 0.80–0.93) and AUC 0.79 (95% CI: 0.70–0.88), respectively [124]. MALAT 1 also has the potential of being a predictive biomarker. Wang et al. determined that enzalutamide resistant prostate cancer cell lines have increased levels of MALAT 1 and SF2 activity, whereas MALAT 1 may enhance AR-V7 splicing by forming a MALAT1-SF2 complex leading to enzalutamide resistance [125]. In addition, Sebastian et al. reported that MALAT 1 might promote prostate cancer bone metastasis through down-regulation of Sost pathway [126]. The diagnostic capability of urinary MALAT 1 was evaluated in a retrospective-prospective study of 434 men [127]. Wang et al. showed that by using the MALAT 1 model, unnecessary biopsy could be safely avoided in up to 46.5% of diagnostic grey zone cohorts where their PSA lies between 4–10 ng/mL [127].

lncRNA SChLAP 1 (Second Chromosome Locus Associated with Prostate 1 or known as LINC00913) has been reported to be highly expressed in patients with prostate cancer after microarray analysis of 960 prostate cancer tissue samples [128]. In addition, Prensner et al. found that SChLAP 1 has higher expression in metastatic progression cases compared to non-metastatic cases showing the potential to be a prognostic marker to distinguish patients who are at high risk of metastatic disease progression [128]. SChLAP promotes cell invasion and metastasis by interfering with gene regulation through the SWI/SNF complex interaction, thereby obtunding the tumor suppressor effects from the complex [128]. Recently, SChLAP 1 has been shown to act as a miRNA sponge, causing the negative regulation of miR-198, thus promoting the MAPK1 pathway [129]. Li Y et al. showed that SChLAP 1 acted inversely with miR-198, in which knockdown of SChLAP 1 significantly up-regulates miR-198 expression [129]. Prensner et al. further validated the expression of SChLAP 1 in prostate cancer tissues as a risk factor for metastatic prostate cancer progression from a large cohort of 1008 patients with prostate cancer post radical prostatectomy [130]. The group showed that SChLAP 1 expression is prognostic for metastatic progression whereby the multivariate modeling independently predicted 10 year metastatic-free survival (OR: 2.45, 95% CI: 1.70–3.53; *p* < 0.0001) [130]. Another study looking at SChLAP 1 expression using an RNA in situ hybridization (RISH) assay reported similar findings of poor outcome in localised stage patients post prostatectomy when SChLAP 1 is highly expressed assessed via RISH (univariate HR = 2.343, *p* = 0.005; multivariate HR = 1.99, *p* = 0.032) [131]. Urinary SChLAP 1 expression was investigated in 230 patients and was shown to mirror that of tissue expression whereby patients with higher urinary SChLAP 1 expression had a higher risk of disease progression [132]. In addition, Wang YH et al. have recently reported that SChLAP 1 is also highly expressed in plasma tumor-derived exosomes of patients with prostate cancer [133]. The group isolated exosomes from the plasma of patients with prostate cancer, BPH and normal controls. SChLAP1 was significantly up-regulated in prostate cancer compared to BPH and normal controls. Furthermore, for the grey zone cohort, exosomal SChLAP 1 plasma expression could distinguish between prostate cancer and BPH with a diagnostic performance of AUC of 0.9898 [133].

FR0348383 is another highly expressed lncRNA initially found during RNA sequencing of 14 matched pairs of prostate cancer tissues and adjacent normal prostate tissues of Chinese patients post prostatectomy, whereby its expression was able to distinguish prostate cancer from BPH [134]. The same group then examined the value of urinary FR0348383 as a diagnostic marker for prostate cancer in 213 patients (94 patients had a PSA between 4–10 ng/mL) and found that the higher the FR0348383 score, the higher the possibility of a positive biopsy (*p* < 0.001) [135]. The FR0348383 score (which is defined as ratio of PSA mRNA and FR0348383 lncRNA level x1000) had an AUC of 0.815, which is superior to PSA (AUC: 0.562), PSA density (AUC: 0.645) and percentage free PSA (AUC: 0.599) in the diagnostic grey zone cohort [135] showing its promising diagnostic potential (Table 3).

### 3.4. Circular RNAs (circRNAs) as Biomarkers for Prostate Cancer

circRNAs are a group of endogenous RNAs, discovered in 1990s, which are produced from backsplicing of exons, introns, or both giving rise to exonic or intronic circRNAs [66]. However, they were initially thought to represent the by-products of splicing error until recently [66,152,153,154], when the appeal of circRNA research was resurrected by Salzman et al. who found a substantial number of circRNAs in malignant and non-malignant cell lines, suggesting that they may be more abundant and of more importance than previously perceived [155]. In 2012, Salzman et al. found an excessive number of RNA transcripts, in which the exons were arranged non-canonically, during an effort to find genomic rearrangements in RNA-seq samples of paediatric acute lymphoblastic leukaemia [155]. These excessive RNA transcripts in malignant and non-malignant cell lines were then validated as circRNA by qualitative PCR (qPCR). Two other groups further developed the methods of circRNA detection by locating specific backsplice junction *de novo* from RNA-seq libraries using bioinformatic analyses which strengthen the foundation of this field [66,156]. Since then, numerous detection methods have emerged with the addition of bioinformatic analyses which enhances the ability to detect and quantify the expression of new circRNAs.

The advancement in NGS has allowed the identification of large numbers of circRNAs [157]. However, the RNA-Seq datasets remain inconsistent due to non-standardization of circRNA enrichment strategies in data generation on top of the relatively low quantity of circRNA compared to linear RNA [158]. Currently, there is no ‘gold standard’ method to validate the algorithm’s performance in circRNA identification [158,159]. Online circRNA databases are available for investigators to use such as CircBase, Circ2Traits and CircInteractome [157,160,161].

circRNAs have been proven to represent a distinct class of ncRNAs, with the 3′ and 5′ ends connected together resulting in the formation of a covalently closed continuous loop, which makes the circRNA resistant to degradation by RNA exonucleases [66,162]. Thus, circRNA is present abundantly in the cytoplasm owing to its stability and the cellular levels may be regulated by either removal by exosomes or endonucleic activity [163]. This triad of stability, abundance and evolutionary preservation between species indicates that circRNA may have a significant regulatory function [164]. In addition, circRNA is expressed in pathological conditions coupled with tissue specificity, which further begs the question of their role in malignancy and other human illness [165]. 

circRNAs have been recently discovered to exhibit a functional role in regulating cancer development and progression [166]. The role of miRNA in cancer pathogenesis on the other hand, is well established [167]. The interest in circRNA as a biomarker was further amplified when circRNAs were discovered to act as miRNA sponges and regulate a specific miRNA target gene responsible for cancer initiation or progression [168]. circRNA may act as miRNA sponges through a competitive endogenous RNA (ceRNA) network, which suggests that specific circRNA can compellingly influence miRNA activity through requisition leading to either up-regulation or down-regulation of miRNA target gene expression [169,170]. Many circRNAs possess miRNA binding sites for single or even multiple miRNAs [168]. Numerousstudies have described the importance of the circRNA-miRNA network in the pathogenesis of cancer (Table 2). Other alternative routes that circRNA may regulate gene expression is by acting as a protein sponge or decoy, for instance circ_Foxo3 can form a complex with p21 and CDK2 preventing cyclin E/CDK2 complex formation, which is necessary for cells to transit from G1 to S phase, thus arresting cell cycle at G1 phase [171].

circRNAs could potentially be an ideal biomarker owing to their universal expression patterns accompanied by unique characteristics such as tissue specificity, stability and evolutionary conservation [165,172,173]. Salzman et al. established that circRNAs not only encompass a substantial fraction of cellular RNA but are also expressed specifically between cell types [165]. This is also reflected by the findings of circRNAs dysregulation across different types of malignancy [174]. The presence of circRNA in human bodily fluid such as saliva, blood and gastric fluid further supports its possibility as a disease biomarker [175,176,177]. Bahn et al. have shown that salivary circRNAs are associated with inflammatory response and intercellular signaling [175]. Memczak et al. found that circRNA can be robustly detected in blood, highlighting the fact that blood circRNA may be a better biomarker candidate than linear RNA [176]. Furthermore, Yan Li et al. demonstrated that circRNA is enriched by at least two-fold and stable in exosomes making it a promising biomarker [178] whereas Shao et al. confirmed the stability of circRNA in gastric fluid despite freeze-thaw and incubation processes [177]. With all these elements combined, circRNA could be an ideal tumor biomarker candidate.

A number of RNA biomarkers have been investigated in prostate cancer; however, there is a scarcity of literature describing the role of circRNA as a biomarker. Kong et al. have identified the up-regulation of circSRAMRCA5 (hsa_circ_0001445) across four prostate cancer cell lines (LNCAP, 22RV1, DU145 and PC-3) compared to a normal prostate cell line (WPMY-1) and in 21 prostate cancer tissue samples compared with corresponding normal prostate tissue [179]. Interestingly, this study also found that circSMARCA5 expression level can be affected by androgens, as circSMARCA5 level increased according to dihydrotestosterone (DHT) level in a dose-dependent manner. Other work demonstrated the possibility of circSMARCA5 to act as a potential pro-oncogenic circRNA in vitro affecting cell cycle and apoptosis [179]. Recently, Dai et al. found that circRNA-MYLK (hsa_circ_0141940) is significantly up-regulated in prostate cancer cell lines (LNCAP, DU145, PC-3 and PC-3MIE8) in comparison to normal prostate cell lines (WPMY-1). The group also found that circRNA-MYLK is highly up-regulated in 17 prostate cancer tissues in comparison to corresponding normal prostate tissues [180]. The effect of circRNA-MYLK on prostate cancer cell proliferation, apoptosis, invasion and migration were further studied. Knockdown of circRNA-MYLK significantly inhibited cell viability, increased apoptotic cell number and decreased the number of invasive migratory prostate cancer cells; however, those effects were reversed in an up-regulated circRNA-MYLK setting. The group further showed that the up-regulation of circRNA-MYLK acted as a miRNA sponge in down-regulating miR29a [180]. miR-29a mimics were shown to reverse the promotive effect of circRNA-MYLK in cell growth, invasion, and migration in PC-3 cells transfected with circRNA-MYLK over-expression vector. This study further strengthens the potential role of circRNA, not only as a diagnostic biomarker but also as a therapeutic biomarker for prostate cancer. Even though there is a paucity of literature describing the role of circRNA in prostate cancer, there is evidence demonstrating the role of circRNA in other malignancies (Table 4).

### 3.5. Small Nucleolar RNAs (snoRNAs) and snoRNA-Derived RNAs (sdRNAs) as Biomarkers for Prostate Cancer

snoRNA is a group of non-coding RNA, which consist of 60–300 nucleotides in length and are further categorised to either C/D box snoRNAs or H/ACA box snoRNAs [197,198]. They interact with ribonucleoproteins (RNPs) to form stable snoRNP particles, which are involved in post-transcriptional modification of ribosomal RNA and small nuclear RNA [199]. Previously, snoRNAs have been considered as housekeeping non-coding molecules involved in ribosomal RNA modification and maturation [200], however, there has been a growing body of evidence that suggest other, additional novel functions [201,202]. Gong J et al. reported that both the expression of snoRNAs and RNPs were generally observed to be up-regulated across multiple malignancies suggestive of a possible synergistic role in their pathogenesis [203]. Furthermore, the group also found that over-expression of RNPs were associated with poor prognosis across multiple tumours types [203].

Dong et al. in 2008 found a homozygous 2 base pair chromosomal deletion of snoRNA U50 gene in 2 of 30 prostate cancer cell lines/xenografts and 9 of 89 patients with localised prostate cancer but none in the 104 healthy controls [204]. The group further confirmed that a homozygous deletion of snoRNA U50 was significantly associated with prostate cancer after analysing 1371 patients with prostate cancer and 1371 healthy controls (OR: 2.9, 95% CI: 1.17–7.21) [204]. This suggests snoRNAs have the ability to act as tumour suppressors and thus have the capacity to act as a therapeutic biomarker.

snoRNA can be further processed to sdRNAs which is abundantly expressed in prostate cancer [82,200,205]. sdRNAs were initially thought to be sequencing artefacts or by-products of snoRNA, however, they are evolutionary conserved and functionally associated with genetic disorders and cancer [82,206,207]. Studies have reported that sdRNA can mimic miRNA-like activity and are involved in an RNA silencing mechanism [208,209]. Martens-Uzunova et al. sequenced all the small RNA of matched normal and prostate cancer samples of patients in different disease stages followed by validation of the expression of snoRNAs (SNORD44, SNORD78, SNORD74 and SNORD81) and sdRNAs in a cohort of 106 patients [205]. The group reported that all snoRNAs and derivate sdRNAs were increased in prostate cancer tissue compared to normal paired controls. In addition, the cohort that developed metastatic disease had significantly (*p* < 0.0001) up-regulated snoRNA (SNORD78) and its derivate sdRNA, thus demonstrating its capability of being a prognostic biomarker [205]. Crea et al. systematically evaluated the prognostic capability as well as the functions of snoRNAs by performing RNA sequencing on paired metastatic and non-metastatic prostate cancer xenografts whereby 21 differentially expressed snoRNAs were found between the two [210]. Twelve of the 21 snoRNAs present in the cBio cancer genomic portal clinical database were selected for validation in two independent cohorts of patients with prostate cancer. The study showed that an up-regulation of SNORA55 is associated with significant shorter relapse free survival after surgery (univariate; median survival 35.35 vs. > 140 months, *p* = 0.015). Tissue SNORA55 expression was also significantly up-regulated in prostate cancer samples compared to BPH samples (mean expression: 8.345 ± 2.555 vs. 1.342 ± 0.3729, *p* = 0.0095). Serum SNORA55 was also investigated in nine brachytherapy treated patients compared to five normal controls and was found to be significantly up-regulated in the prostate cancer cohort [210]. The group also showed that prostate cancer cells proliferation and migration is significantly obtruded when SNORA55 was silenced [210]. Thus, SNORA55 has the potential of being a prognostic and therapeutic biomarker. In addition, snoRNAs as potential biomarker have also been investigated in other malignancies (Table 5).

### 3.6. Transfer RNAs (tRNAs) and tRNA-Derived RNA Fragments (tRFs) as Biomarkers for Prostate Cancer

tRNA is a group of non-coding RNA which are 76–90 nucleotides in length [216] and play a role in protein translation. They can also regulate gene expression and participate in cellular processes such as cell proliferation [217]. tRNA can be further processed to smaller RNA species known as tRFs detectable through next generation sequencing [218,219]. tRFs can be categorised based on size into either stress-induced tRFs (30–35 nucleotides) or 5′end- and 3′-derived tRFs (approximately 20 nucleotides) [219,220]. Lee et al. sequenced the expression of small RNA in human prostate cancer cell lines and found that tRFs are abundantly expressed second only to miRNAs [218]. The group further selected the most frequently expressed tRF in cell lines, known as tRF-1001, and performed siRNA-mediated knockdown to evaluate its biological importance [218]. tRF-1001 knockdown cell lines were shown to be less viable, with cell proliferation impairment accompanied by reduction in DNA synthesis and accumulation of cells in G2 phase of cell cycle. This further supported the notion that tRFs are not incidental tRNAs degradation by-products [218]. Olvedy et al. recently examined the expression of tRFs in paired prostate cancer tissues at different stages of disease and normal adjacent prostate tissues [221]. A total of 598 tRFs were deregulated in prostate cancer samples compared to paired normal prostate tissue with 5′-derived tRFs representing the majority of up-regulated tRFs. The group further selected 6 tRFs for analysis and found 4 tRFs were up-regulated and 2 tRFs were down-regulated. These were further validated in tissue samples of 2 cohorts of patients with prostate cancer (cohort 1 = 65 samples and cohort 2 = 104 samples). tRF-544 expression was significantly down-regulated in patients with recurrent prostate cancer and also in patients with a Gleason score above 7 or higher stage suggestive of tumour aggressiveness or late stage association [221]. In contrast, tRF-315 was significantly up-regulated in recurrent prostate cancer and high-grade tumours. The group also reported that the ratio of tRFs-315 to tRF-544 is able to distinguish high grade prostate cancer from low grade disease and high tRFs-315 to tRF-544 ratio correlates with inferior progression free survival [221]. Thus, tRNAs has the potential of being a prognostic marker.

### 3.7. Piwi-Interacting RNAs (piRNAs)as Biomarkers for Prostate Cancer

piRNA is a group of small non-coding RNA which consist of 29–30 nucleotides [222]. By binding to PIWI proteins, piRNAs-PIWI complex exhibit the ability to regulate genes, exert transposon silencing and play a role in germline stem cell maintenance [223,224]. In 2014, piRNAs were found to be involved in mRNA regulation [225]. Evidence of a role for piRNAs in cancer pathogenesis is emerging [226,227,228,229]. Martinez VD et al. recently examined the human piRNAs transcriptomes from malignant and non-malignant tissues of 11 organs including prostate. The group found that piRNA expression is tissue specific in particular for prostate tissues. In general, piRNAs expression was found to be higher in malignant tissue compared to non-malignant tissues and is able to distinguish between the two [230]. piRNA has been recently shown to be a potential prognostic biomarker in colorectal cancer [227], however there is a paucity of literature available on piRNAs as a biomarker for prostate cancer.

## 4. Conclusions

Dr. Richard J. Ablin first discovered PSA in 1970 and for the last two decades since the FDA approved its use, PSA remains the standard biomarker for diagnosis, screening and monitoring of prostate cancer despite having many flaws. Moving beyond PSA, the only RNA biomarker approved, in 2012, for use in a clinical setting as a diagnostic decision tool is the lncRNA PCA3. PCA3 shows improved specificity but still lacks the sensitivity to be an independent diagnostic tool. Even though we have added a myriad of therapies to the armamentarium, there is a need to develop effective biomarkers that will identify patients at an earlier stage of disease, detect disease progression accurately, tailor treatment according to response and act as therapeutic targets. For instance, one of the most successful RNA biomarker tests is the Oncotype DX in breast cancer, which has been validated rigorously in multiple independent prospective studies [231,232,233]. The test is able to accurately select patients with early stage hormone sensitive HER2 negative breast cancer who will benefit from adjuvant chemotherapy, which encompasses approximately 15% of this population, thus sparing the remainder from unnecessary treatment [234]. The treatment paradigm of early stage breast cancer has shifted as a result of Oncotype DX where a reduction in the over-treatment of low risk patients with breast cancer can be observed. Similarly, over-treatment is one of the major clinical issues in prostate cancer management, especially when treatment can result in catastrophic quality of life. As mentioned above, the Oncotype DX test in prostate cancer is currently being appraised in a multi-center clinical trial setting (ClinicalTrials.gov Identifier: NCT03502213). In this review, we have delineated the developmental transition of RNA as potential biomarkers for prostate cancer from cell line models and tissue samples to liquid biopsies. Multiple RNA biomarker combinations may improve sensitivity and specificity in diagnostic and prognostic values compared to a single RNA biomarker. RNA as a potential biomarker has many advantages, as it can be detected in bodily fluid, which enables minimally invasive diagnostics. In addition, RNA expression level is closely associated with the gene regulation machinery which reflects the functional state of the biological system. Thus, measuring RNA biomarkers delivers the most direct path for assessment of the cell’s functional state. The absence of uniform and robust technologies can be considered one of the obvious pitfalls in RNA biomarker innovations through to clinical application. However, with the advancement in NGS, the potential of RNA biomarker research will continue to unfold and become a crucial tool, which promises to surpass PSA which further sets the rhythm of personalized precision oncology.

## Figures and Tables

**Table 1:** CRPC treatments with an overall survival (OS) benefit.

Treatments	Trial	OS Benefit (Months)	Year	Author
Docetaxel + Prednisolone	TAX 327	2.4	2004	[9]
Sipuleucel-T	IMPACT	4.1	2010	[21]
Cabazitaxel + Prednisolone	TROPIC	2.4	2010	[22]
Enzalutamide	AFFIRM	4.8	2012	[11]
Abiraterone Acetate + Prednisolone	COU-AA-301	4.6	2012	[23]
Alpha-Radium 223	ALSYMPCA	3.6	2013	[24]

**Table 2:** miRNA expression in prostate cancer and associated clinical outcomes.

miRNA Expression	Samples	Associated Clinical Outcomes	Author
miR-96 ↑	Tissue	Increase biochemical recurrence Decreased recurrence-free survival.	[80]
miR-96 ↑	Tissue	Poor overall survival Increase tumor aggressiveness.	[81]
(miR-96-5p, miR-183-5p) ↑*, (miR-145-5p, miR-221-5p) ↓*	Tissue	Distinguish prostate cancer from normal control. Increase tumor aggressiveness. Increase metastatic risk.Poor overall survival.	[83]
miR-221 ↓	Tissue	Increase biochemical recurrence.	[90]
(miR-21, miR-22, miR-141) ↑	Plasma	Distinguish prostate cancer from normal control. Distinguished between metastatic and localized disease.	[91]
(miR-141, miR-375) ↑	Serum, Tissue	Associated with high risk prostate cancer (Gleason score and lymphnode involvement).	[96]
(miR-20a, miR-21, miR-145, miR-221) ↑	Plasma	Associated with high risk prostate cancer (CAPRA and D’Amico score).	[100]
(miR-107, miR-574-3p) ↑	Urine	Distinguish prostate cancer from normal control.	[103]
(miR-200b, miR-200c) ↑	plasma	Associated with high risk prostate cancer (Gleason score, PSA, metastasis).	[105]

↓ = decrease, ↑ = increase, * part of miQ score.

**Table 3:** Potential lncRNAs as prostate cancer biomarker.

lncRNAs	Expression	Sample	Potential Biomarker	Author
PCA 3	↑	tissues/urine	diagnostic/therapeutic	[114,117,118,121,122]
MALAT 1	↑	tissues/plasma	diagnostic/predictive	[112,123,124,125,127]
SChLAP 1	↑	tissues/plasma/urine	diagnostic/prognostic	[128,130,132,133]
FR0348383	↑	tissues/urine	diagnostic	[134,135]
PCAT1	↑	cell lines/tissues	therapeutic	[136,137]
CCAT2	↑	tissues	prognostic	[138]
CTBP1-AS	↑	tissues	prognostic	[139]
DRAIC	↓	cell lines	prognostic	[140]
HCG11	↓	tissues	prognostic	[141]
LINC01296	↑	cell lines/tissues	prognostic	[142]
LincRNA-p21	↓	cell lines	prognostic	[143]
LncRNA-ATB	↑	tissues	prognostic	[144]
LOC440040	↑	cell lines/tissues	prognostic	[145]
NEAT1	↑	cell lines/tissues	prognostic	[146]
PCAT14	↑ (early)/ ↓ (late) disease	tissues	prognostic	[147]
PCGEM1	↑	tissues	prognostic	[148,149]
TRPM2-AS	↑	tissues	prognostic	[150]
UCA1	↑	tissues	prognostic	[151]

↓ = decrease, ↑ = increase.

**Table 4:** circRNA-miRNA pathway in cancer.

Tumour	Associated circRNA	Target miRNA	Hypothesized miRNA Function	Regulatory Role of circRNA on miRNA	circRNA Expression in Tumor	Potential circRNA Biomarker	Author
Bladder carcinoma	circTCF25	miR-103a-3p/miR-107	Tumor supressor-miR	Negative	↑	na	[181]
Bladder carcinoma	cir-ITCH	miR-17/miR-224	Onco-miR	Negative	↓	prognostic	[182]
Bladder carcinoma	circRNA-Cdr1as	miR-135a	Onco-miR	Negative	↓	na	[183]
Breast carcinoma	circ-ABCB10	miR-1271	Tumor supressor-miR	Negative	↑	na	[184]
Breast carcinoma	circRNA-000911	miR-449a	Onco-miR	Negative	↓	prognostic	[185]
Colorectal carcinoma	hsa_circ_000984	miR-106b	Onco-miR	Positive	↑	na	[186]
Colorectal carcinoma	hsa_circ_001569	miR-145	Tumor supressor-miR	Negative	↑	na	[187]
Colorectal carcinoma	circHIPK3	miR-7	Tumor supressor-miR	Negative	↑	prognostic	[188]
Esophageal carcinoma	cir-ITCH	miR-7/miR-17/miR-214	Onco-miR	Negative	↓	na	[189]
Gastric carcinoma	circPVT1	miR-125 family	Tumor supressor-miR	Negative	↑	prognostic	[190]
Hepatocellular carcinoma	circMTO1	miR-9	Onco-miR	Negative	↓	prognostic	[191]
Hepatocellular carcinoma	circRNA-Cdr1as	miR-7	Tumor supressor-miR	Negative	↑	na	[192]
Lung carcinoma	hsa_circ_0013958	miR-134	Tumor supressor-miR	Negative	↑	diagnostic	[193]
Lung carcinoma	cir-ITCH	miR-7/miR-214	Onco-miR	Negative	↓	na	[194]
Osteosarcoma	circUBAP2	miR-143	Tumor supressor-miR	Negative	↑	prognostic	[195]
Prostate carcinoma	circMYLK	miR-29a	Tumor supressor-miR	Negative	↑	diagnostic/ therapeutic	[180]
Renal carcinoma	circHIAT1	miR-195-5p/miR-29a-3p/miR-29c-3p	Tumor supressor-miR	Positive	↓	therapeutic	[196]

na = not available, all sample used was tissues except * = tissues and plasma, ↓ = decrease, ↑ = increase.

**Table 5:** Potential snoRNA as prostate cancer biomarker.

Tumour	snoRNAs	Expression	Sample	Potential Biomarker	Hypothesized RNAs function	Author
Prostate carcinoma	SNORA55	↑	tissues/serum	prognostic/therapeutic	Oncogene	[210]
Prostate carcinoma	snoRNA U50 *	↓	tissues/serum	diagnostic	Tumor-suppressor	[204]
Prostate carcinoma	SNORD78	↑	tissues	prognostic	na	[205]
Lung carcinoma	SNORA42	↑	tissues	prognostic/ therapeutic	Oncogene	[211]
Lung carcinoma	SNORD33/ SNORD66/ SNORD76	↑	plasma	diagnostic	Oncogene	[212]
Hepatocellular carcinoma	SNORD113-1	↓	tissues	prognostic/ therapeutic	Tumor-suppressor	[213]
Glioblastoma	SNORD76	↓	tissues	prognostic	Tumor-suppressor	[214]
Gastric carcinoma	SNORD105b	↑	tissues	prognostic/ therapeutic	Oncogene	[215]

↓ = decrease, ↑ = increase, * = homozygous 2 base pair deletion in U50 gene, na = not available.
